# Learning stochastic dynamics and predicting emergent behavior using transformers

**DOI:** 10.1038/s41467-024-45629-w

**Published:** 2024-02-29

**Authors:** Corneel Casert, Isaac Tamblyn, Stephen Whitelam

**Affiliations:** 1 Molecular Foundry, Lawrence Berkeley National Laboratory, 1 Cyclotron Road, Berkeley, CA 94720 USA; 2 Department of Physics and Astronomy, Ghent University, 9000 Ghent, Belgium; 3 Cash App, Block, Toronto, ON M5A 1J7 Canada; 4 Vector Institute for Artificial Intelligence, Toronto, ON M5G 1M1 Canada; 5 Department of Physics, University of Ottawa, Ottawa, ON K1N 6N5 Canada

**Keywords:** Phase transitions and critical phenomena, Statistical physics, Computer science

## Abstract

We show that a neural network originally designed for language processing can learn the dynamical rules of a stochastic system by observation of a single dynamical trajectory of the system, and can accurately predict its emergent behavior under conditions not observed during training. We consider a lattice model of active matter undergoing continuous-time Monte Carlo dynamics, simulated at a density at which its steady state comprises small, dispersed clusters. We train a neural network called a transformer on a single trajectory of the model. The transformer, which we show has the capacity to represent dynamical rules that are numerous and nonlocal, learns that the dynamics of this model consists of a small number of processes. Forward-propagated trajectories of the trained transformer, at densities not encountered during training, exhibit motility-induced phase separation and so predict the existence of a nonequilibrium phase transition. Transformers have the flexibility to learn dynamical rules from observation without explicit enumeration of rates or coarse-graining of configuration space, and so the procedure used here can be applied to a wide range of physical systems, including those with large and complex dynamical generators.

## Abstract (web summary)

Learning the dynamics governing a simulation or experiment usually requires coarse graining or projection, as the number of transition rates typically grows exponentially with system size. The authors show that transformers, neural networks introduced initially for natural language processing, can be used to parameterize the dynamics of large systems without coarse graining.

## Introduction

Learning the dynamics governing a simulation or experiment is a difficult task, because the number of possible dynamical transitions increases exponentially with the physical size of the system. For large systems, these transitions are too numerous be enumerated explicitly, and what is usually done is to coarse-grain or project a system’s dynamical degrees of freedom into a subspace small enough to be learned explicitly^1–8^. Here we present a dynamics-learning method that does not require projection or coarse-graining, even for large systems. We show that a recently-introduced neural network called a transformer^9^, popular in the fields of natural-language processing and computer vision^10–14^, can express a general dynamics without the need for coarse-graining over the model’s degrees of freedom or choosing a sub-space of dynamical processes to learn. It can be trained offline, i.e., by observation only^15^, to learn the dynamical rules of a model, even when those rules are numerous and nonlocal. Forward-propagated trajectories of the trained transformer can then be used to reproduce the behavior of the observed model, and to predict its behavior when applied to conditions not seen during training.

Previous work has shown that it is possible to learn the rules of deterministic dynamics, such as deterministic cellular automata^16–18^, or of stochastic dynamics for small state spaces, using maximum-likelihood estimation on the rates of the generator^19,20^. Similar methods have been used to learn the form of intermolecular potentials that influence the dynamical trajectories of particle systems^21–25^. Machine learning and symbolic regression have been used to rediscover Newton’s formula for the gravitational force from trajectories of the solar system^26^. The accurate prediction of fluid dynamics and turbulent flows has been achieved with physics-informed neural networks^27–29^. Several approaches exist in which high-dimensional dynamical systems are approximated by lower-dimensional ones, such as Markov-state models^1–3^. In some cases the coarse-graining procedures used to produce such models involve variational methods^4^ and neural networks^5^. Coarse-graining methods have also been used to learn molecular dynamics^8^, and to obtain deterministic hydrodynamic equations from stochastic trajectories of active matter, allowing for the extraction of hydrodynamic transport coefficients^6,7^. Our work complements these approaches by showing that it is possible to learn the dynamical rules of stochastic systems without explicit enumeration of rates or coarse-graining of configuration space, thereby allowing treatment of large and complex systems. From the observation of a single dynamical trajectory a transformer can identify how many classes of move exist and what are their rates, providing physical insight into the dynamics and allowing it to be simulated in new settings, where new phenomena can be discovered.

We focus on the case of a lattice model of active matter, simulated using continuous-time Monte Carlo dynamics^30^ (in the Supplemental Information (SI) we show that the transformer can be used to treat a second class of model, one realization of which has nonlocal dynamical rules.). We allow the transformer to know that the rates for this dynamics are independent of time, and that possible moves consist of single particles rotating in place or translating one lattice site at a time (both restrictions can be relaxed within our framework). However, we do not allow the transformer to know the rates for each move, and, because each rate could in principle depend on the state of the entire system, explicit enumeration of rates would require a generator with many more than 10^100^ entries for the system size considered. From observation of a single trajectory of the model, carried out at a density at which its steady state comprises small, dispersed clusters, the transformer learns that particle moves fall into a small number of classes, and accurately determines the associated rates. Forward-propagated trajectories of the trained transformer at the training density reproduce the model’s behavior. Moreover, forward-propagated trajectories of the transformer carried out at densities higher than that used in training exhibit motility-induced phase separation (MIPS)^31–35^. The details of this phase separation match those seen using the original model, although that information was not available to the transformer during training. The trained transformer is therefore able to accurately extrapolate a learned dynamics to predict the existence and details of an emergent phenomenon that it had not previously observed. Given that the transformer is expressive enough to represent a nonlocal dynamics, these results indicate the potential of such devices to learn dynamical rules and study emergent phenomena from observations of dynamical trajectories in a wide variety of settings.

**Fig. 1:**
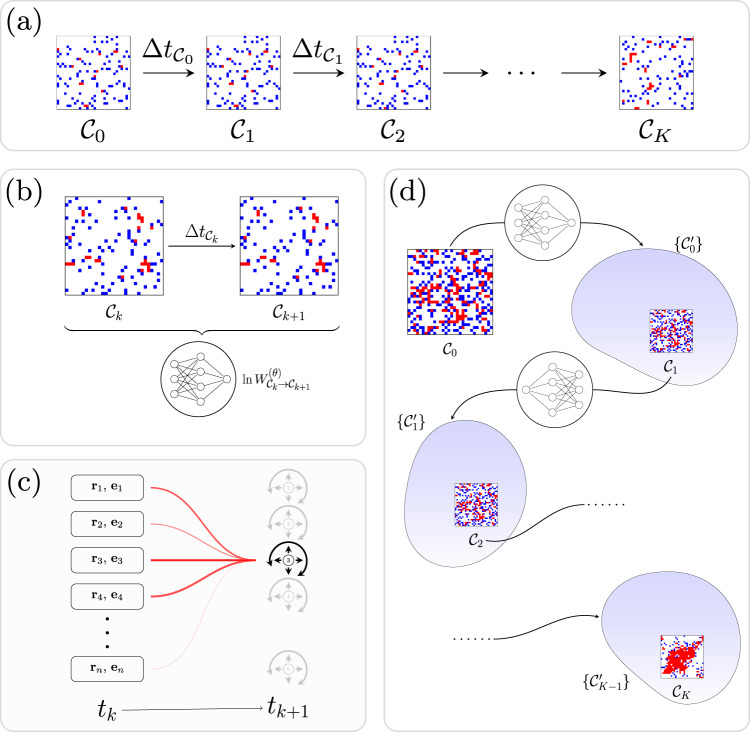
Schematic of our dynamics-learning procedure. **a** We are provided with a trajectory *ω*, a time series of configurations, and wish to learn the dynamics that created it. For the lattice-based active-matter model studied here, red or blue indicates a particles whose orientation vector points toward an occupied or empty site, respectively. **b** We parameterize a general dynamics using a neural network called a transformer. Rates connecting configurations depend on the weights of the transformer, which are adjusted during training in order to maximize the log-likelihood with which it would have generated *ω*. **c** The transformer receives the position and orientation of all particles, and must calculate the transition rates to translate or rotate each particle. To do so, it must learn which interactions affect these rates (line thickness denotes attention given to each particle), and their numerical values. **d** Once trained, the neural-network dynamics can be forward-propagated to generate new trajectories, even under conditions not observed in *ω*. The transformer calculates the rates for all possible transitions ${{{{{{{{\mathcal{C}}}}}}}}}_{k}\to \{{{{{{{{{\mathcal{C}}}}}}}}}_{k}^{{\prime} }\}$, represented by the blue blobs, at each step.

Imagine that we are given a dynamical trajectory *ω* of total time *T*. The trajectory starts in configuration (microstate) ${{{{{{{{\mathcal{C}}}}}}}}}_{0}$, and visits *K* additional configurations ${{{{{{{{\mathcal{C}}}}}}}}}_{k}$ (Fig. 1a). In configuration ${{{{{{{{\mathcal{C}}}}}}}}}_{k}$ it is resident for time $\Delta {t}_{{C}_{k}}$. Schematically,

$$\omega={{{{{{{{\mathcal{C}}}}}}}}}_{0}\mathop{\longrightarrow }\limits^{\Delta {t}_{{{{{{{{{\mathcal{C}}}}}}}}}_{0}}}{{{{{{{{\mathcal{C}}}}}}}}}_{1}\mathop{\longrightarrow }\limits^{\Delta {t}_{{{{{{{{{\mathcal{C}}}}}}}}}_{1}}}\cdots {{{{{{{{\mathcal{C}}}}}}}}}_{K-1}\mathop{\longrightarrow }\limits^{\Delta {t}_{{{{{{{{{\mathcal{C}}}}}}}}}_{K-1}}}{{{{{{{{\mathcal{C}}}}}}}}}_{K}\mathop{\longrightarrow }\limits^{\Delta {t}_{K}}{{{{{{{{\mathcal{C}}}}}}}}}_{K},$$

where $\Delta {t}_{K}\equiv T-\mathop{\sum }\nolimits_{k=0}^{K-1}\Delta {t}_{{{{{{{{{\mathcal{C}}}}}}}}}_{k}}$. We are told that *ω* was generated by a dynamics whose rates ${W}_{{{{{{{{\mathcal{C}}}}}}}}\to {{{{{{{{\mathcal{C}}}}}}}}}^{{\prime} }}^{\star }$ for passing between configurations ${{{{{{{\mathcal{C}}}}}}}}$ and ${{{{{{{{\mathcal{C}}}}}}}}}^{{\prime} }$ we do not know. We will call this unknown dynamics the original dynamics. Here we show it is possible to efficiently learn the original dynamics offline, i.e., solely by observation of *ω*. We start by constructing a synthetic dynamics, which consists of a set of allowed configuration changes $\{{{{{{{{\mathcal{C}}}}}}}}\to {{{{{{{{\mathcal{C}}}}}}}}}^{{\prime} }\}$ (which must include those observed in *ω*) and associated rates ${W}_{{{{{{{{\mathcal{C}}}}}}}}\to {{{{{{{{\mathcal{C}}}}}}}}}^{{\prime} }}^{({{{{{{{\boldsymbol{\theta }}}}}}}})}$. Without prior knowledge of the system we should allow the rates for these moves to depend, in principle, on the entire configuration of the system. The number of possible rates grows exponentially with system size, and so treating a system of appreciable size requires the use of an expressive parameterization of the synthetic dynamics. Here we parameterize the rates ${W}_{{{{{{{{\mathcal{C}}}}}}}}\to {{{{{{{{\mathcal{C}}}}}}}}}^{{\prime} }}^{({{{{{{{\boldsymbol{\theta }}}}}}}})}$ of the synthetic dynamics using the weights ***θ*** of a neural network.

One way to learn the original dynamics is to propagate the synthetic dynamics and alter its parameters ***θ*** until the dynamical trajectories it generates resemble *ω*. One drawback of this approach is that original and synthetic dynamics are stochastic, and so comparison of trajectories can be made only in a statistical sense, potentially requiring the generation of many synthetic trajectories at each stage of training. In addition, a comparison of this nature would require the introduction of additional order parameters, different combinations of which may result in different outcomes of training. Instead, we train the synthetic dynamics by maximizing the log-likelihood ${U}_{\omega }^{({{{{{{{\boldsymbol{\theta }}}}}}}})}$ with which it would have generated *ω*^19^. We consider continuous-time Monte Carlo dynamics, in which case

1 $${U}_{\omega }^{({{{{{{{\boldsymbol{\theta }}}}}}}})}=\mathop{\sum }\limits_{k=0}^{K-1}\left(\ln {W}_{{{{{{{{{\mathcal{C}}}}}}}}}_{k}\to {{{{{{{{\mathcal{C}}}}}}}}}_{k+1}}^{({{{{{{{\boldsymbol{\theta }}}}}}}})}-\Delta {t}_{{{{{{{{{\mathcal{C}}}}}}}}}_{k}}{R}_{{{{{{{{{\mathcal{C}}}}}}}}}_{k}}^{({{{{{{{\boldsymbol{\theta }}}}}}}})}\right) -\Delta {t}_{K}{R}_{{{{{{{{{\mathcal{C}}}}}}}}}_{K}}^{({{{{{{{\boldsymbol{\theta }}}}}}}})};$$

see Methods. Training proceeds by adjusting the parameters ***θ*** of the neural network until ${U}_{\omega }^{({{{{{{{\boldsymbol{\theta }}}}}}}})}$ no longer increases; see Fig. 1(b). For a sufficiently long trajectory *ω*, the dynamics that maximizes ${U}_{\omega }^{({{{{{{{\boldsymbol{\theta }}}}}}}})}$ is the original dynamics, ${W}_{{{{{{{{\mathcal{C}}}}}}}}\to {{{{{{{{\mathcal{C}}}}}}}}}^{{\prime} }}^{\star }$. The synthetic dynamics obtained in this way—the learned dynamics—is then the best approximation to the original dynamics that our choice of allowed configuration changes and method of training allows. We focus here on the case of continuous-time Monte Carlo dynamics and lattice configurations, but the method can be straightforwardly adapted to other scenarios. Working with another class of dynamics (e.g., Langevin dynamics) requires defining a replacement for the trajectory log-likelihood Eq. (1). Working with off-lattice configurations requires an appropriate parameterization of the possible microscopic moves, but the transformer itself is not restricted to taking lattice-based configurations as inputs.

## Results and discussion

The original dynamics we consider is a lattice model of active matter simulated using continuous-time Monte Carlo^30^ (we also consider a lattice model of a supercooled liquid in the SI, see Supplementary Figs. S1–S5). It consists of a two-dimensional periodic square lattice of size *L*^2^, occupied by *n* volume-excluding particles. Each particle *α* ∈ {1, …, *n*} possesses a unit orientation vector **e**_*α*_ that points toward one of the four neighboring sites. The orientation vector of each particle rotates *π*/2 clockwise or counter-clockwise with rate *D*. A particle moves to a vacant adjacent lattice site with rate *v*_+_ if it points toward that lattice site, and with rate *v*_0_ otherwise. The steady state of this model depends on the particle density *ϕ* = *n*/*L*^2^. At small values of *ϕ*, typical configurations consist of small clusters of particles. Upon increasing *ϕ*, for sufficiently large *v*_+_, the system undergoes the nonequilibrium phase transition called MIPS. We shall show that the existence of this phase transition can be deduced by observation of a single trajectory obtained at a value of *ϕ* at which MIPS is not present.

We introduce a general synthetic dynamics using a neural-network architecture called a transformer^9^. We allow the transformer to know only that the dynamics is time-independent and consists of single-particle translations and rotations, though these restrictions can be lifted within this framework. In microstate ${{{{{{{\mathcal{C}}}}}}}}$, the transformer represents the transition rates ${W}_{{{{{{{{\mathcal{C}}}}}}}}\to {{{{{{{{\mathcal{C}}}}}}}}}^{{\prime} }}^{({{{{{{{\boldsymbol{\theta }}}}}}}})}$ to each of the microstates ${{{{{{{{\mathcal{C}}}}}}}}}^{{\prime} }$ connected to ${{{{{{{\mathcal{C}}}}}}}}$ through translation or rotation of a single particle (Fig. 1c). The transformer learns which particle interactions are relevant to each of these moves, and what their rates are. To train the transformer we perform gradient descent on its weights using backpropagation in order to maximize the log-likelihood ${U}_{\omega }^{({{{{{{{\boldsymbol{\theta }}}}}}}})}$, Eq. (1), with which it would have generated *ω*. This trajectory is of length *T* = 5 × 10^3^, using a 30 × 30 lattice, with parameters $\phi=0.12\overline{4},\;{v}_{+}=10,\;{v}_{0}=1$, and *D* = 0.1. MIPS is not present at these parameter values; see Fig. 3.

During training we operate the transformer in one of two modes. In Mode 1, the transformer freely predicts $\ln {W}_{{{{{{{{\mathcal{C}}}}}}}}\to {{{{{{{{\mathcal{C}}}}}}}}}^{{\prime} }}^{({{{{{{{\boldsymbol{\theta }}}}}}}})}$ for each possible transition. In Mode 2, the transformer assigns each transition to one of an integer number ${N}_{W}^{({{{{{{{\boldsymbol{\theta }}}}}}}})}$ of classes, and a second neural network assigns a value $\ln {W}_{{{{{{{{\mathcal{C}}}}}}}}\to {{{{{{{{\mathcal{C}}}}}}}}}^{{\prime} }}^{({{{{{{{\boldsymbol{\theta }}}}}}}})}$ to each class. ${N}_{W}^{({{{{{{{\boldsymbol{\theta }}}}}}}})}$ is a hyperparameter that constrains the complexity of the learned dynamics, and provides a measure of the number of distinct classes of move (or processes) present in the original dynamics: the maximum value of ${U}_{\omega }^{({{{{{{{\boldsymbol{\theta }}}}}}}})}$ obtained under training increases with ${N}_{W}^{({{{{{{{\boldsymbol{\theta }}}}}}}})}$ up to a value ${N}_{W}^{\star }$. The value ${N}_{W}^{\star }$ provides insight into the structure of the generator of the original dynamics, signaling, for instance, the presence of translational invariance. In Methods, additional details of the architectures of both types of neural-network dynamics and their optimization are provided. We have used lattice models in this paper, but the transformer architecture can be directly applied to off-lattice models in any dimension.

**Fig. 2:**
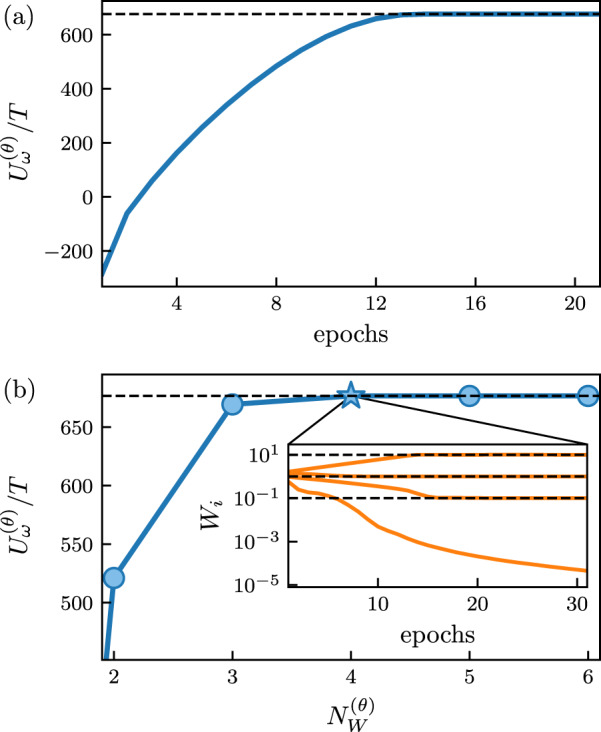
Learning the dynamics of the lattice active-matter model. **a** Training of a transformer in Mode 1 (unrestricted rates) to maximize the log-likelihood ${U}_{\omega }^{({{{{{{{\boldsymbol{\theta }}}}}}}})}$, Eq. (1), of the training trajectory *ω*. The horizontal black line denotes the value of the path weight associated with the original model. **b** Dependence of ${U}_{\omega }^{({{{{{{{\boldsymbol{\theta }}}}}}}})}$ for a transformer trained in Mode 2, in which it is asked to identify ${N}_{W}^{({{{{{{{\boldsymbol{\theta }}}}}}}})}$ distinct classes of move. This procedure allows us to identify the existence of ${N}_{W}^{\star }=4$ distinct rates. Inset: Evolution of the rates during training in Mode 2, with ${N}_{W}^{({{{{{{{\boldsymbol{\theta }}}}}}}})}=4$. The horizontal black lines denote the values of the rates in the original dynamics.

In Fig. 2a we show the results of training in Mode 1. The trajectory log-likelihood ${U}_{\omega }^{({{{{{{{\boldsymbol{\theta }}}}}}}})}$ increases with the number of observations (epochs) of the trajectory *ω*, and converges to the value ${U}_{\omega }^{\star }$ that is obtained using the original dynamics. This value, not available to the transformer during training, indicates that the learned transition rates *W* ^(***θ***)^ are numerically very close to those of the original dynamics, *W* ^⋆^. In Fig. 2b we show the results of training in Mode 2, for several values of ${N}_{W}^{({{{{{{{\boldsymbol{\theta }}}}}}}})}$. These results show that ${N}_{W}^{\star }=4$, indicating that the transformer has correctly learned the degree of complexity of the original model, whose dynamical rules are translationally invariant and consist of 4 distinct rates. The inset to Fig. 2b shows the evolution with training time of the values of the 4 rates, compared with their values in the original model.

During training we did not assume that the dynamical rules are local, nor that some processes (those that violate volume exclusion) are suppressed. The transformer was able to learn both things. If we know that interactions are of finite range then such knowledge can be used to reduce the number of transformer parameters required to learn dynamics (see the SI). Transformers can also learn long-ranged interactions if they are present, which we illustrate in Supplementary Figs. S6 and S7 in the SI. We also note that learned rates for forbidden processes (inset Fig. 2b) are small and decrease with training time, but are not exactly zero: the result is that in forward-propagated trajectories a small fraction of particles can experience overlaps. If volume exclusion is suspected then it can be imposed directly. In addition, with Monte Carlo methods it is possible to determine that the rate of a forbidden process is exactly zero, even given a finite-length training trajectory; see Table S1 in the SI.

**Fig. 3:**
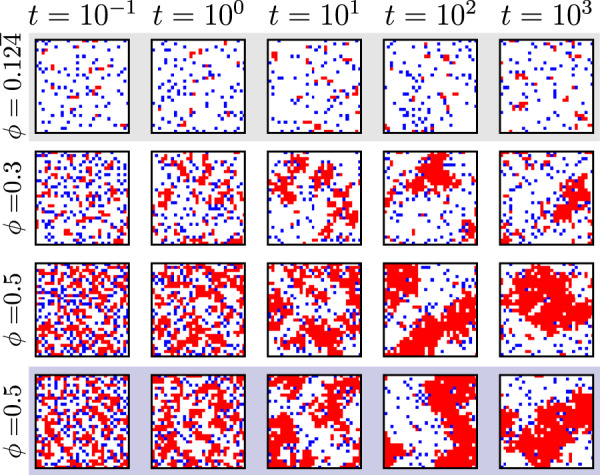
Trajectories of the lattice active-matter model generated using the dynamics learned by the transformer. The top row shows time-ordered snapshots of a trajectory generated at density $\phi=0.12\overline{4}$, the value used during training. The two middle rows use densities *ϕ* = 0.3 and *ϕ* = 0.5; here, motility-induced phase separation can be seen. For comparison, the bottom row shows a trajectory generated with the original dynamics at *ϕ* = 0.5.

In Fig. 3 we show that trajectories generated by the trained transformer can be used to determine the existence of a nonequilibrium phase transition not seen during training. We randomly initialize a configuration at a chosen density *ϕ* and propagate the transformer dynamics for fixed time *T* (see Fig. 1d and Methods). At the training density $\phi=0.12\overline{4}$, the model’s steady state consists of small clusters, but trajectories generated by the transformer at larger values of *ϕ* show MIPS: the transformer has therefore predicted this emergent phenomenon.

**Fig. 4:**
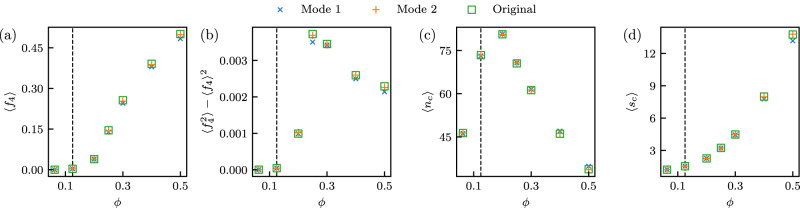
Quantitative comparison of the learned and original dynamics at different densities. **a** Time-averaged fraction of particles with four neighboring occupied sites, *f*_4_, as a function of density *ϕ*, averaged over 10 trajectories of length 10^4^, generated using a transformer trained in Mode 1 (crosses) and Mode 2 (plusses). Training was done only at $\phi=0.12\overline{4}$ (vertical dashed line). Squares denote results obtained using the original dynamics. The remaining panels have the same format and show (**b**) the variance of *f*_4_, (**c**) the number of clusters *n*_*c*_, and (**d**) the averaged cluster size *s*_*c*_. Angle brackets denote time averages.

In Fig. 4 we quantify the details of this phase separation. We measure the fraction of particles with four neighboring occupied sites *f*_4_, and the variance of that quantity, as well as the number of clusters *n*_c_ and the average cluster size *s*_c_. The time averages of these observables are shown as a function of *ϕ* for trajectories obtained with the transformer, both in Mode 1 and Mode 2. For comparison, we show the same quantities from trajectories generated using the original dynamics. The agreement between original and learned dynamics is good, and slightly better using Mode 2, indicating that the transformer, trained under conditions for which no phase separation is observed (see the vertical line in the figure), has predicted the existence and details of a non-equilibrium phase transition (we have verified that we can similarly learn the dynamics at high density and accurately predict the behavior at low density).

We have shown that the stochastic dynamics of a many-body system can be efficiently determined using machine-learning tools developed for language processing. A neural network called a transformer can function as an expressive ansatz for the generator of a many-body dynamics, for systems large enough that its possible rates are too numerous to represent explicitly. For instance, for the lattice model of active matter considered here, a 30 × 30 lattice at density *ϕ* = 0.1 admits $$\left(\begin{array}{c}900\\ 90\end{array}\right) \sim 1{0}^{125}$$ arrangements of particles. Each particle takes 1 of 4 rotational states, can move in 4 directions and undergo 2 types of rotation, meaning that there are in principle $\sim {10}^{180}$ possible rates. Trained on this model, the transformer learns its dynamics, correctly identifying its local and translationally-invariant nature, and the numerical values of the associated rates. Forward-propagated trajectories of the transformer, carried out at higher densities than that observed during training, show MIPS. The details of this nonequilibrium phase transition predicted by the transformer agree with those of the original model. Our work shows that it is possible to learn the dynamical rules of stochastic systems without explicit enumeration of rates or coarse-graining of configuration space, complementing existing papers on learning dynamics and pointing the way to the treatment of large and complex systems.

## Methods

### Derivation of the path weight of a continuous-time Monte Carlo dynamics

Consider a dynamical trajectory *ω* of total time *T*, which starts in configuration ${{{{{{{{\mathcal{C}}}}}}}}}_{0}$ and visits *K* additional configurations ${{{{{{{{\mathcal{C}}}}}}}}}_{k}$. Schematically,

$$\omega={{{{{{{{\mathcal{C}}}}}}}}}_{0}\mathop{\longrightarrow }\limits^{\Delta {t}_{{{{{{{{{\mathcal{C}}}}}}}}}_{0}}}{{{{{{{{\mathcal{C}}}}}}}}}_{1}\mathop{\longrightarrow }\limits^{\Delta {t}_{{{{{{{{{\mathcal{C}}}}}}}}}_{1}}}\cdots {{{{{{{{\mathcal{C}}}}}}}}}_{K-1}\mathop{\longrightarrow }\limits^{\Delta {t}_{{{{{{{{{\mathcal{C}}}}}}}}}_{K-1}}}{{{{{{{{\mathcal{C}}}}}}}}}_{K}\mathop{\longrightarrow }\limits^{\Delta {t}_{K}}{{{{{{{{\mathcal{C}}}}}}}}}_{K},$$

where $\Delta {t}_{{C}_{k}}$ is the time spent in configuration ${{{{{{{{\mathcal{C}}}}}}}}}_{k}$ and $\Delta {t}_{K}\equiv T-\mathop{\sum }\nolimits_{k=0}^{K-1}\Delta {t}_{{{{{{{{{\mathcal{C}}}}}}}}}_{k}}$.

The trajectory *ω* was generated by a continuous-time Monte Carlo dynamics (the original dynamics), whose rates whose rates ${W}_{{{{{{{{\mathcal{C}}}}}}}}\to {{{{{{{{\mathcal{C}}}}}}}}}^{{\prime} }}^{\star }$ for passing between configurations ${{{{{{{\mathcal{C}}}}}}}}$ and ${{{{{{{{\mathcal{C}}}}}}}}}^{{\prime} }$ are unknown. In order to learn the original dynamics, we introduce a new continuous-time Monte Carlo model called the synthetic dynamics. The synthetic dynamics consists of a set of allowed configuration changes $\{{{{{{{{\mathcal{C}}}}}}}}\to {{{{{{{{\mathcal{C}}}}}}}}}^{{\prime} }\}$ (which must include those observed in *ω*) and associated rates ${W}_{{{{{{{{\mathcal{C}}}}}}}}\to {{{{{{{{\mathcal{C}}}}}}}}}^{{\prime} }}^{({{{{{{{\boldsymbol{\theta }}}}}}}})}$. Rates are parameterized by a vector ***θ*** = {*θ*_1_, …, *θ*_*N*_} of *N* numbers (in the main text these numbers corresponds to the weights of the transformer). We train the synthetic dynamics by maximizing the log-likelihood ${U}_{\omega }^{({{{{{{{\boldsymbol{\theta }}}}}}}})}$ with which it would have generated *ω*. To calculate ${U}_{\omega }^{({{{{{{{\boldsymbol{\theta }}}}}}}})}$ we start by considering the portion

2 $${{{{{{{{\mathcal{C}}}}}}}}}_{k}\mathop{\longrightarrow }\limits^{\Delta {t}_{{{{{{{{{\mathcal{C}}}}}}}}}_{k}}}{{{{{{{{\mathcal{C}}}}}}}}}_{k+1}$$

of *ω*, which involves a transition ${{{{{{{{\mathcal{C}}}}}}}}}_{k}\to {{{{{{{{\mathcal{C}}}}}}}}}_{k+1}$ and a residence time $\Delta {t}_{{{{{{{{{\mathcal{C}}}}}}}}}_{k}}$. The probability with which the synthetic dynamics would have generated the transition ${{{{{{{{\mathcal{C}}}}}}}}}_{k}\to {{{{{{{{\mathcal{C}}}}}}}}}_{k+1}$ is

3 $${W}_{{{{{{{{{\mathcal{C}}}}}}}}}_{k}\to {{{{{{{{\mathcal{C}}}}}}}}}_{k+1}}^{({{{{{{{\boldsymbol{\theta }}}}}}}})}/{R}_{{{{{{{{{\mathcal{C}}}}}}}}}_{k}}^{({{{{{{{\boldsymbol{\theta }}}}}}}})},$$

where ${R}_{{{{{{{{{\mathcal{C}}}}}}}}}_{k}}^{({{{{{{{\boldsymbol{\theta }}}}}}}})}\equiv {\sum }_{{{{{{{{{\mathcal{C}}}}}}}}}^{{\prime} }}{W}_{{{{{{{{{\mathcal{C}}}}}}}}}_{k}\to {{{{{{{{\mathcal{C}}}}}}}}}^{{\prime} }}^{({{{{{{{\boldsymbol{\theta }}}}}}}})}$, the sum running over all transitions allowed from ${{{{{{{{\mathcal{C}}}}}}}}}_{k}$. The probability density with which the synthetic dynamics would have chosen the associated residence time $\Delta {t}_{{{{{{{{{\mathcal{C}}}}}}}}}_{k}}$ is

4 $${R}_{{{{{{{{{\mathcal{C}}}}}}}}}_{k}}^{({{{{{{{\boldsymbol{\theta }}}}}}}})}{{{{{{{{\rm{e}}}}}}}}}^{-\Delta {t}_{{{{{{{{{\mathcal{C}}}}}}}}}_{k}}{R}_{{{{{{{{{\mathcal{C}}}}}}}}}_{k}}^{({{{{{{{\boldsymbol{\theta }}}}}}}})}}$$

The product of transition- and residence-time factors is

5 $${W}_{{{{{{{{{\mathcal{C}}}}}}}}}_{k}\to {{{{{{{{\mathcal{C}}}}}}}}}_{k+1}}^{({{{{{{{\boldsymbol{\theta }}}}}}}})}{{{{{{{{\rm{e}}}}}}}}}^{-\Delta {t}_{{{{{{{{\mathcal{C}}}}}}}}}{R}_{{{{{{{{{\mathcal{C}}}}}}}}}_{k}}^{({{{{{{{\boldsymbol{\theta }}}}}}}})}}\equiv {p}_{{{{{{{{{\mathcal{C}}}}}}}}}_{k}}.$$

Noting that the probability of the final portion of the trajectory, ${{{{{{{{\mathcal{C}}}}}}}}}_{K}\mathop{\longrightarrow }\limits^{\Delta {t}_{K}}{{{{{{{{\mathcal{C}}}}}}}}}_{K}$, is

6 $$1-\int\nolimits_{0}^{\Delta {t}_{K}}{{{{{{{\rm{d}}}}}}}}\tau \,{R}_{{{{{{{{{\mathcal{C}}}}}}}}}_{k}}^{({{{{{{{\boldsymbol{\theta }}}}}}}})}{{{{{{{{\rm{e}}}}}}}}}^{-{R}_{{{{{{{{{\mathcal{C}}}}}}}}}_{k}}^{({{{{{{{\boldsymbol{\theta }}}}}}}})}\tau }={{{{{{{{\rm{e}}}}}}}}}^{-\Delta {t}_{K}{R}_{{{{{{{{{\mathcal{C}}}}}}}}}_{K}}^{({{{{{{{\boldsymbol{\theta }}}}}}}})}}\equiv {p}_{K},$$

the log-likelihood with which the synthetic dynamics would have generated *ω* is

7 $${U}_{\omega }^{({{{{{{{\boldsymbol{\theta }}}}}}}})} 	=\ln \left({p}_{K}\mathop{\prod }\limits_{k=0}^{K-1}{p}_{{{{{{{{{\mathcal{C}}}}}}}}}_{k}}\right)\\ 	=\mathop{\sum }\limits_{k=0}^{K-1}\left(\ln {W}_{{{{{{{{{\mathcal{C}}}}}}}}}_{k}\to {{{{{{{{\mathcal{C}}}}}}}}}_{k+1}}^{({{{{{{{\boldsymbol{\theta }}}}}}}})}-\Delta {t}_{{{{{{{{{\mathcal{C}}}}}}}}}_{k}}{R}_{{{{{{{{{\mathcal{C}}}}}}}}}_{k}}^{({{{{{{{\boldsymbol{\theta }}}}}}}})}\right) -\Delta {t}_{K}{R}_{{{{{{{{{\mathcal{C}}}}}}}}}_{K}}^{({{{{{{{\boldsymbol{\theta }}}}}}}})}.$$

The sum in (7) is taken over the trajectory *ω*, i.e., over all configuration changes and corresponding residence times (we note that working with the probability ${R}_{{{{{{{{{\mathcal{C}}}}}}}}}_{k}}^{({{{{{{{\boldsymbol{\theta }}}}}}}})}{{{{{{{{\rm{e}}}}}}}}}^{-\Delta {t}_{{{{{{{{{\mathcal{C}}}}}}}}}_{k}}{R}_{{{{{{{{{\mathcal{C}}}}}}}}}_{k}}^{({{{{{{{\boldsymbol{\theta }}}}}}}})}}\Delta {t}_{{{{{{{{{\mathcal{C}}}}}}}}}_{k}}$ for the residence time gives rise to an additional term $\mathop{\sum }\nolimits_{k=0}^{K-1}\Delta {t}_{{{{{{{{{\mathcal{C}}}}}}}}}_{k}}$ in (7) that does not depend on the choice of synthetic dynamics and may be omitted without consequence). To train the synthetic dynamics we adjust its parameters ***θ*** until (7) no longer increases.

### Neural-network architecture and training

The neural network used to treat the active-matter model described in the main text (and the models described in the SI) is a transformer^9^, originally developed for language processing. We have opted for this architecture for two main reasons: (1) a transformer does not introduce a bias toward interaction ranges when learning the dynamics most likely to have generated the observed trajectory, and (2) a transformer can efficiently learn symmetries and locality in the interaction rules. This ability stands in contrast to other neural-network architectures such as fully-connected neural networks or convolutional neural networks. A convolutional neural network, for instance, is parameterized using small kernels which slide along the input configuration. This means that in order to capture long-range interactions in the data, we need to apply many convolutional layers successively, and the choice of neural-network depth introduces a bias on the range of interactions we want to learn. Likewise, the weight sharing of the kernels in the convolutional layer introduces a bias toward translational invariance of the interaction rules. A fully-connected neural network does consider interactions between all elements of the system, but because it lacks meaningful positional information it can not efficiently learn whether interactions are local, or whether there are symmetries present in the data.

A transformer possesses an attention mechanism—explained below—that allows it to learn which parts of a configuration are relevant for a particular process. This generality ensures that it is not biased toward learning local interactions, as is the case for e.g., convolutional neural networks, but can efficiently learn locality if needed.

The first step in calculating the transition rates is a learned representation of the current state of the system. We first embed particle positions and orientations as *d*_h_-dimensional vectors using trainable weight matrices; *d*_h_ is a hyperparameter controlling the expressivity of our neural-network model. For the positional embedding of the active matter model, we map the *x*- and *y*-coordinate of each particle to a vector of size *d*_h_/2 using a weight matrix, and then concatenate these representations. For computational efficiency, we do not use the empty sites. Instead, the transformer must learn which neighboring sites are occupied for each particle through the positional embedding. We do not impose the boundary conditions of our lattice models; the transformer has to learn these through its positional embedding.

We then sum the representations of the position and spin for each particle, which serve as the input to the first layer of the transformer. Next, we calculate the attention matrix for the configuration using scaled dot-product attention^9^. This means that we construct a query, key, and value vector for each input particle through a linear transformation. We match the query vector of each particle against all the keys through a dot product, resulting in an attention score for all combinations of keys with the query. These scores are then normalized, and the output of the attention layer is obtained through a sum of the value vectors of every particle, each weighted by the attention score. As a result, we obtain a *d*_h_-dimensional vector for each particle, containing a weighted sum of features of all other particles (the weighting being a measure of the attention paid to each particle). This mechanism can be applied in parallel, where multiple key, query, and value combinations (“heads”) are produced at each stage, allowing to attend to different properties of the input sentence simultaneously.

These vectors are then processed using fully-connected neural networks, where we apply the same fully-connected network to each vector. We apply this alternating process of attention and application of fully-connected neural networks *n*_l_ times. The final output of these transformations is used to calculate the transition rate for each possible particle update (a particle rotation or translation for the active-matter model).

Training in Mode 1, the rates are obtained by applying a fully-connected neural network to the output vectors of the transformer. We apply the same network for each particle. This fully-connected neural network has one output node for each possible particle update, the value of $\ln W$ assigned to the corresponding transition. Training in Mode 2, we first classify the transformer’s output vectors using a fully-connected neural network with *N*_*W*_ output nodes and a softmax activation function, again for each possible particle update. The class with the highest probability is sent, as a one-hot vector, to another fully-connected neural network with one output node, which calculates the value of $\ln W$ for each of the *N*_*W*_ classes. Picking the highest-probability class is not a differentiable operation, and so we use a straight-through estimator to obtain the gradients to optimize these neural networks^36^.

The results in this paper were obtained with the hyperparameters *d*_h_ = 64 and *n*_l_ = 2. We used the AdaBelief optimizer^37^ with a learning rate of 10^−4^ to optimize the transformer’s weights. To obtain a baseline for the trajectory log-likelihood ${U}_{\omega }^{({{{{{{{\boldsymbol{\theta }}}}}}}})}$, we first train a Mode 1 neural-network dynamics on the provided trajectory. For efficiency we train for several epochs on smaller sections of the trajectory; during the final stages of training we use the entire trajectory to obtain more accurate gradients of the trajectory log-likelihood. Next, we train a Mode 2 neural-network dynamics to gain insight into the model’s generator. We initialize the first layers of the neural network (the embedding and transformer layers) with the weights obtained with the Mode 1 dynamics, which leads to much faster convergence.

We have here assumed that the dynamics are independent of time, and the only possible moves are single-particle translations and rotations. We note that these assumptions may also be lifted: time could be used as an additional input to the neural network, and collective updates could be achieved using an encoder-decoder architecture as used in language translation^9^.

The transformer architecture can by construction be applied to configurations consisting of a different number of particles (much like transformers used in natural language processing can be used to model sentences with a different number of words). The transformer receives as input a sequence of *n* particles (i.e., their position and their state), and returns the transition rates for each particle in the input sequence. This means that we can naturally apply the trained transformer to lattice configurations of the active matter model at the same system size, but at a different particle density than seen during training. In order to provide accurate results at a different density, the transformer has to have learned an accurate representation of how particles interact with one another through its positional embedding.

### Supplementary information

Supplementary Information

## Data Availability

Training trajectories can be generated using the code in Ref. ^38^.

## Code availability

Training code and a tutorial for learning dynamics can be found in ref. ^38^.
