# Supplementary material for: Learning stochastic dynamics and predicting emergent behavior using transformers
Source: Nat Commun. 2024 Feb 29;15:1875. doi: 10.1038/s41467-024-45629-w (PMC10904374; doi:10.1038/s41467-024-45629-w)
Supplement: Supplementary file 1 — Supplementary Information [file 41467_2024_45629_MOESM1_ESM.pdf]

Supplemental Information to "Learning stochastic dynamics and predicting emergent behavior using transformers"

Corneel Casert,[1,2,*] Isaac Tamblyn,[3,4,5,†] and Stephen Whitelam[1,‡]

[1] *Molecular Foundry, Lawrence Berkeley National Laboratory, 1 Cyclotron Road, Berkeley, CA 94720, USA*
[2] *Department of Physics and Astronomy, Ghent University, 9000 Ghent, Belgium*
[3] *Cash App, Block, Toronto, ON M5A 1J7, Canada*
[4] *Vector Institute for Artificial Intelligence, Toronto, ON M5G 1M1, Canada*
[5] *Department of Physics, University of Ottawa, Ottawa, ON K1N 6N5, Canada*

In sections S1, S2 and S3, we illustrate the training procedure described in the main text in situations of increasing complexity.

S1. LEARNING A LOCAL DYNAMICS WITH KNOWLEDGE OF ITS LOCALITY

In this section we consider an original dynamics whose rules are local, and assume that we know that its rules are local. The learning procedure then amounts to identifying the correct numerical values for the rates of each local process. This is a conceptually simple case, but worth considering because it illustrates the precision with which rates can be learned, the fact that it is possible to learn the existence of forbidden processes, and also demonstrates some of the convenient features of learning dynamics offline, without propagating new trajectories.

We consider the original dynamics to be the one-dimensional Fredrickson-Andersen (FA) model with periodic boundary conditions [39]. The FA model is a lattice model of a supercooled liquid whose dynamical rules give rise to slow relaxation and complex space-time behavior [40, 41]. On each site of a lattice lives a binary spin that can be down (0) or up (1), intended to model immobile and mobile regions of a supercooled liquid. The dynamical rules for the model are nearest-neighbor ones: spins can only flip if at least one of their nearest neighbors is up; if so, down spins flip up with rate c, and up spins flip down with rate $1-c$. We choose $c = 0.3$, giving the rates shown in Table S1. We use the shorthand 001, 101, etc. to denote the 8 possible configuration changes of a nearest-neighbor dynamics: each triplet indicates the process in which the central spin changes state. Thus 011 indicates the process 011 $\to$ 001, while 000 indicates the process 000 $\to$ 010, etc. The rates for the processes 000 and 010 in the FA model are zero, a feature responsible for the model's complex dynamical behavior.

We start by generating a single FA model trajectory ω of length $T = 10^6$, using a model with 15 lattice sites. This is the trajectory from which we attempt to learn the rates of the model that generated it. A segment of this trajectory of length $T/500$ is shown in Fig. S1(a).

process	true rate	learned rate
000	0	0
001	0.3	0.302028
010	0	0
011	0.7	0.699833
100	0.3	0.300031
101	0.3	0.301362
110	0.7	0.697800
111	0.7	0.702803

TABLE S1. Comparison of the true FA model rates and those learned from the trajectory shown in Fig. S1(a).

To construct the synthetic dynamics we make the assumption that only local processes are allowed, i.e. that only one spin at a time can flip. We also assume that the dynamical rules of the model are independent of time. In this section we further assume that the dynamical rules are local, and that these rules are translationally invariant. The synthetic dynamics constrained in this way contains 8 parameters $\boldsymbol{\theta} = \{\theta_1, \dots, \theta_i, \dots, \theta_8\}$ that correspond to the rates of the 8 processes shown in Table S1.

To find the rates that maximize $U_\omega^{(\boldsymbol{\theta})}$ we proceed as follows. We initialize the rates θ_i by choosing them to be random numbers uniformly distributed on $(0, 1]$, and then apply the following Monte Carlo algorithm. At each step of the learning procedure we propose new parameters

$$\theta_i \to \max\left(0, \theta_i + \mathcal{N}(0, \sigma^2)\right), \tag{S1}$$

and accept the proposal if the value of $U_\omega^{(\boldsymbol{\theta})}$ increases or remains the same. This Monte Carlo algorithm is equivalent, for small values of the proposal-size parameter σ, to noisy clipped gradient ascent on the function $U_\omega^{(\boldsymbol{\theta})}$, described (for $\theta_i > 0$) by the Langevin equation [42]

$$\frac{\mathrm{d}\theta_i}{\mathrm{d}n} = \frac{\sigma}{\sqrt{2\pi}} \frac{1}{|\nabla U_\omega^{(\boldsymbol{\theta})}|} \frac{\partial U_\omega^{(\boldsymbol{\theta})}}{\partial \theta_i} + \eta_i(n). \tag{S2}$$

Here n is the number of steps of the learning algorithm, ∇ is the N-dimensional gradient in the coordinates $\boldsymbol{\theta}$, and η

* ccasert@lbl.gov
† isaac.tamblyn@uottawa.ca
‡ swhitelam@lbl.gov

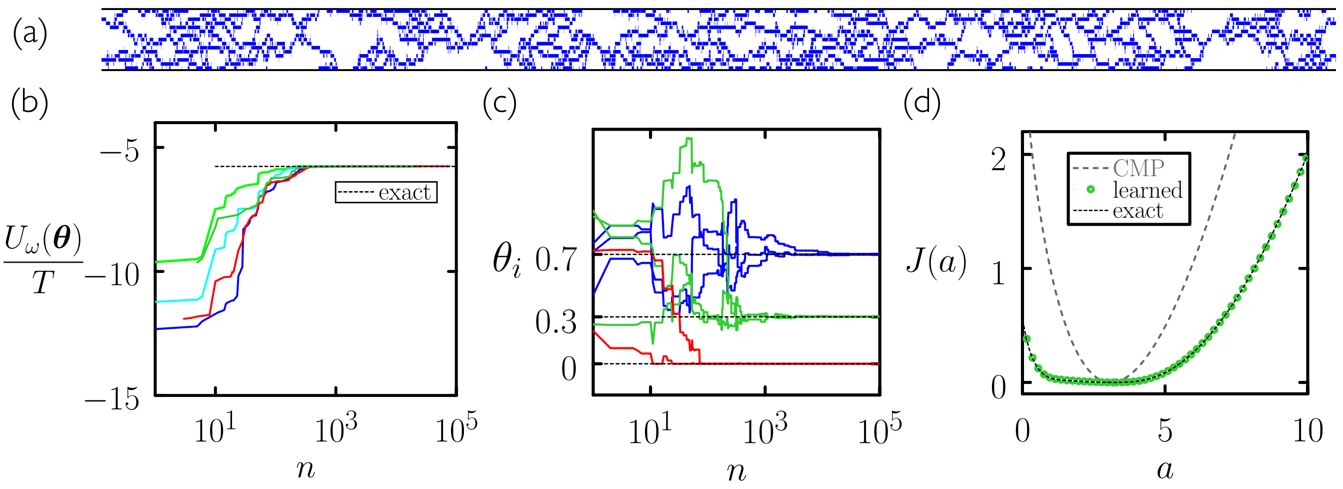

FIG. S1. (a) Portion of the FA model trajectory ω from which we attempt to learn the FA model dynamical rules. Space is vertical, time is horizontal, and `1`s and `0`s are blue and white, respectively. (b) Log-likelihood $U_\omega^{(\boldsymbol{\theta})}$ divided by trajectory length T) with which 5 distinct trained synthetic models realize the trajectory shown in panel (a), as a function of the number of training steps n. (c) Rates for one of the trained synthetic dynamics as a function of n. Rates are color-coded according to their values in the original model: blue, green, and red rates have values in the original model of 0.7, 0.3, and 0, respectively. (d) The large-deviation rate function calculated from forward propagation of 100 distinct trained synthetic models (green) compared with the rate function of the true FA model (black).

is a Gaussian white noise with zero mean and covariance $\langle \eta_i(n)\eta_j(n')\rangle = \sigma^2\delta_{ij}\delta(n-n')/2$. We set $\sigma = 0.05$.

We carried out 100 independent learning simulations, each begun from different random initial rates θ_i, and each trained on the single trajectory ω. Each simulation converged, within about 10^3 steps, to the same value of $U_\omega^{(\boldsymbol{\theta})}$; we show 5 examples in Fig. S1(b) (colored lines). This value is equal to $U_\omega^{(\boldsymbol{\theta})}$ evaluated using the true rates of the FA model (black dashed line), although that information was not available to the algorithm during training. The rates produced by one learning simulation are shown in Fig. S1(c) and Table S1: the learned rates are numerically close to those of the FA model.

Notably, the learning process has correctly identified that the rates `000` and `010` of the model used to produce the trajectory of Fig. S1(a) are exactly zero. Observing that neither transition occurs in a trajectory of finite length allows us only to bound the rates with which those processes occur. The learning procedure has done better, determining that vanishing rates lead to the largest attainable value of $U_\omega^{(\boldsymbol{\theta})}$, and has therefore identified the existence of forbidden processes in the FA model.

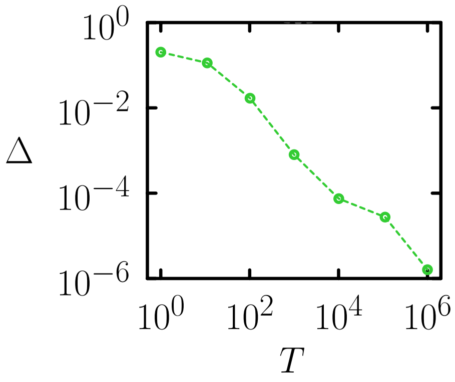

FIG. S2. A measure of the error between original and learned rates, Eq. (S3), as a function of the length T of the training trajectory ω.

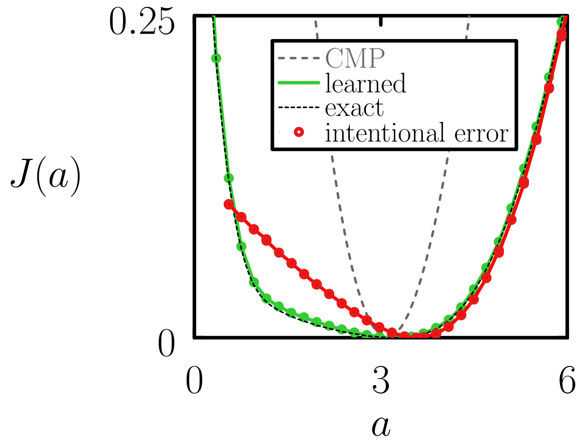

FIG. S3. As Fig. S1(d), but now the red curve results from calculations in which the value 10^{-2} was added to the learned rates `000` and `010` in order to introduce an intentional error. The result – substantially different to the exact answer – shows that comparing the fluctuations of learned and true dynamics is a discriminating test of the learning process, and highlights the precision of that process.

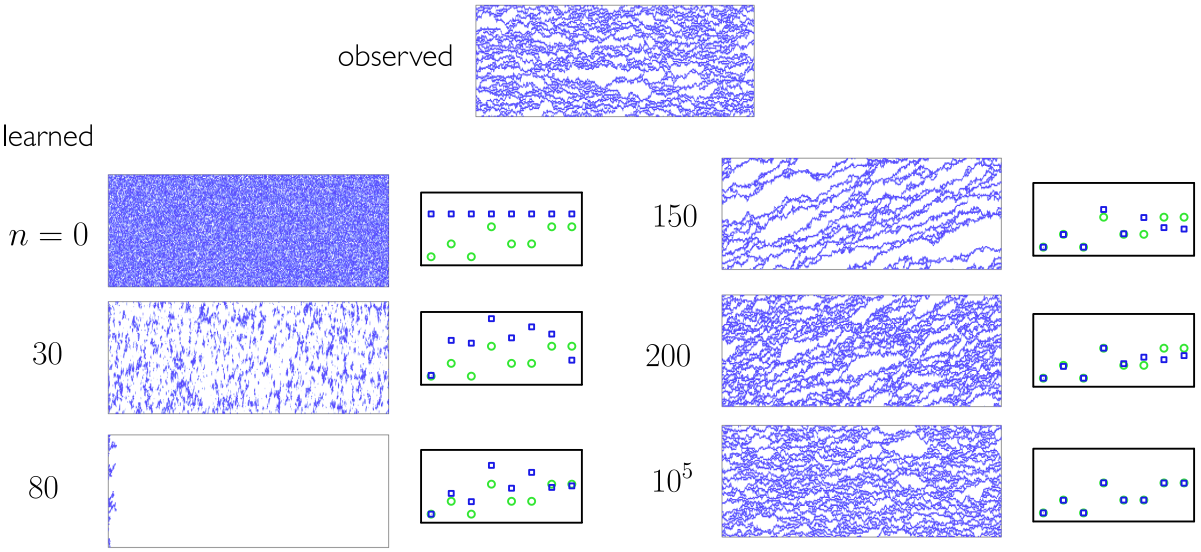

FIG. S4. Comparison of the true FA model dynamics (top) with the forward-propagated dynamics of the synthetic models produced after n steps of training. Small panels show the 8 learned rates (blue squares) and true rates (green circles).

The non-vanishing rates shown in Fig. S1(c) and Table S1 are close to those of the FA model, but not identical. A natural question to ask is how well the learned models can approximate the dynamics generated by the original one. Doing so requires the introduction of order parameters. A popular order parameter for the FA model is the *activity* $a = A/T$, the number of configuration changes A per unit time T [43, 44]. Forward-propagated trajectories of duration $T = 10^6$ of the learned models indeed have the same typical activity as the FA model trajectory, $a_0 \approx 3.2$.

However, a more discriminating measure of similarity is to compare the fluctuations of the learned and original dynamics. Fluctuations of the activity can be characterized by the dynamical large-deviation rate function $J(a) = \lim_{T\to\infty} -T^{-1} \ln \rho_T(A)$, a measure of the logarithmic probability $\ln \rho_T(A)$ of observing, for a trajectory of length T, a particular value of A [45, 46]. We used the VARD method described in Refs. [47, 48] to calculate $J(a)$ for the learned dynamics (we used the neural-network ansatz described in [47]), with each of the 100 learned models constrained to produce a different value of a. Values of $J(a)$ calculated in this way are shown as green circles in Fig. S1(d); they closely approximate the exact rate function of the FA model (black dashed line), which we calculated by numerically diagonalizing the model's rate matrix [46].

The numerical similarity between $J(a)$ of the original and learned models indicates that the precision indicated in Table S1 and in panels (a) and (b) of Fig. S1 is sufficient to produce an essentially exact description of the behavior of the original model, at least as far as the activity is concerned. (In Fig. S3 we show results in which we have made an intentional error by adding 10^{-2} to the learned rates `000` and `010`; in that case there is a clear discrepancy between the learned and true rate functions.)

This comparison also indicates that there is sufficient information in ω to calculate the likelihood with which the original dynamics would produce trajectories never seen in ω. The Conway-Maxwell-Poisson (CMP) function shown in gray in Fig. S1(d) is an upper bound on $J(a)$ inferred by sampling trajectories containing *only* typical configurations [47, 49], such as those seen in Fig. S1(a). The large discrepancy between the CMP bound and the true rate function indicates that rare trajectories of the FA model are dominated by configurations very different to those seen in ω, and therefore never observed during training. Nonetheless, rates inferred from observation of ω can be used to calculate the probability with which long trajectories containing these previously unseen, rare configurations will be observed.

The precision of learning increases with the length of the trajectory ω. In Fig. S2 we show

$$\Delta \equiv N^{-1} \sum_{i=1}^{N} (\theta_i - \theta_i^\star)^2 \,, \tag{S3}$$

the mean-squared difference between the $N = 8$ rates of the FA model $\theta_i^\star$ and the rates θ_i learned from an FA model trajectory ω of length T (a single trajectory is used for each value of T). The precision of the learning process increases with T over the range shown.

We end this section by highlighting a notable feature of the "offline" learning process. During training, no trajectories of the synthetic dynamics are propagated. Given a synthetic dynamics we propose a change of its parameters, calculate the likelihood with which that dynamics *would* have generated the original trajectory ω, and accept the proposal if $U_\omega^{(\boldsymbol{\theta})}$ does not decrease. Some of the proposals accepted in this way would have been rejected

had we trained by comparing ω with forward-propagated trajectories of the synthetic dynamics. In Fig. S4 we propagate some of the synthetic models produced after n steps of training (here we consider an FA model of 100 sites). Next to each trajectory we display a panel comparing the learned rates (blue squares) and true rates (green circles). Here, synthetic rates were initially set to unity. As with the smaller FA model, the training procedure converges to a good approximation of the true rates, and again the rates of the forbidden transitions `000` and `010` are correctly identified to be zero. In general terms the synthetic trajectories look increasingly like those of the FA model as n increases, but there are some notable exceptions. At step $n = 80$ we encounter an absorbing state of all `0`s. This configuration is not accessible to the FA model from any configuration with at least one up spin, and if we were to train by comparison of trajectories generated by original- and synthetic dynamics then this synthetic dynamics would be rejected. However, such a comparison is never made during offline training, and after additional training steps the synthetic dynamics converges to the true dynamics.

S2. LEARNING A LOCAL DYNAMICS *WITHOUT* KNOWLEDGE OF ITS LOCALITY

In Section S1 we assumed that ω was generated by a dynamics whose rules are local and translationally invariant, allowing us to define a synthetic dynamics with few parameters. If we relax these restrictions then the number of possible rates increases exponentially with system size, and so direct representation of the rates of the synthetic dynamics becomes impractical for large systems. Instead, we can express a general synthetic dynamics using a neural network [50], which here we take to be a transformer.

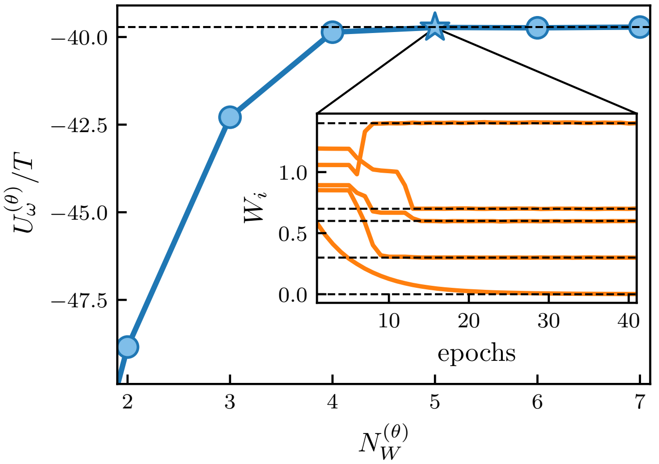

FIG. S5. Training a transformer in Mode 2 to maximize the log-likelihood $U_\omega^{(\boldsymbol{\theta})}$ with which it would have generated a trajectory of an FA model with 100 lattice sites. The value of $U_\omega^{(\boldsymbol{\theta})}$ is maximized if $N_W^{\boldsymbol{\theta}} \geq 5$, which is equal to the number of distinct rates of the original model. The horizontal black line denotes the exact value. Inset: Evolution of the rates during training, for the case $N_W^{(\boldsymbol{\theta})} = 5$. The horizontal black lines denote the values of the rates in the original dynamics.

We now relax the assumptions of locality and translational invariance in its interaction rules, and so the transformer must determine which features of the configuration are relevant to each process (here we use the version of the FA model whose rates are proportional to the number of nearest neighbors in the up state). We generate a training trajectory ω of length $T = 10^6$ using a model with $N = 100$ lattice sites and rate parameter $c = 0.3$. For each configuration of the trajectory the transformer must calculate the rate of flipping each spin, and therefore has to represent $N \times 2^N$ possible transition rates.

We first trained a transformer in Mode 1 (making no restrictions on the number of distinct rate values), and observed that the transformer has the capacity to represent the transition rates for this large state space: the trajectory log-likelihood obtained with the synthetic dynamics rapidly converges to that obtained with the original dynamics. To gain further insight into the generator of the observed trajectory we trained a transformer in Mode 2 on the same trajectory. In Mode 2, the number of distinct rates is limited to $N_W^{(\boldsymbol{\theta})}$, a model hyperparameter. The transformer now must assign each transition to one of $N_W^{(\boldsymbol{\theta})}$ classes, and a second neural network determines the rate for each of these classes.

In Fig. S5, we show how the optimized trajectory log-likelihood depends on the value of $N_W^{(\boldsymbol{\theta})}$. As the number of distinct rates is increased, the trajectory log-likelihood increases until $N_W^{(\boldsymbol{\theta})} \geq 5$, at which point it remains constant. The small number of rates required to model these dynamics tells us that the original dynamics is translationally invariant. We also gain insight into the number of distinct processes present in the original dynamics (because multiple processes can have the same transition rate, this value is a lower bound on the number of distinct processes). In this case, the value $N_W^\star = 5$ is equal to the number of rates in the original dynamics; we compare the exact and learned rates in the inset of Fig. S5.

While restricting the interactions to each spin's nearest neighbors limited the number of rates in Section S1 such that they can be modeled explicitly, prior knowledge of the interactions can also be built into our neural-network framework. For instance, if a maximum interaction range is assumed, local attention [51] can be used which limits the attention calculation to a fixed number of neighboring sites, reducing the computational cost and accelerating convergence.

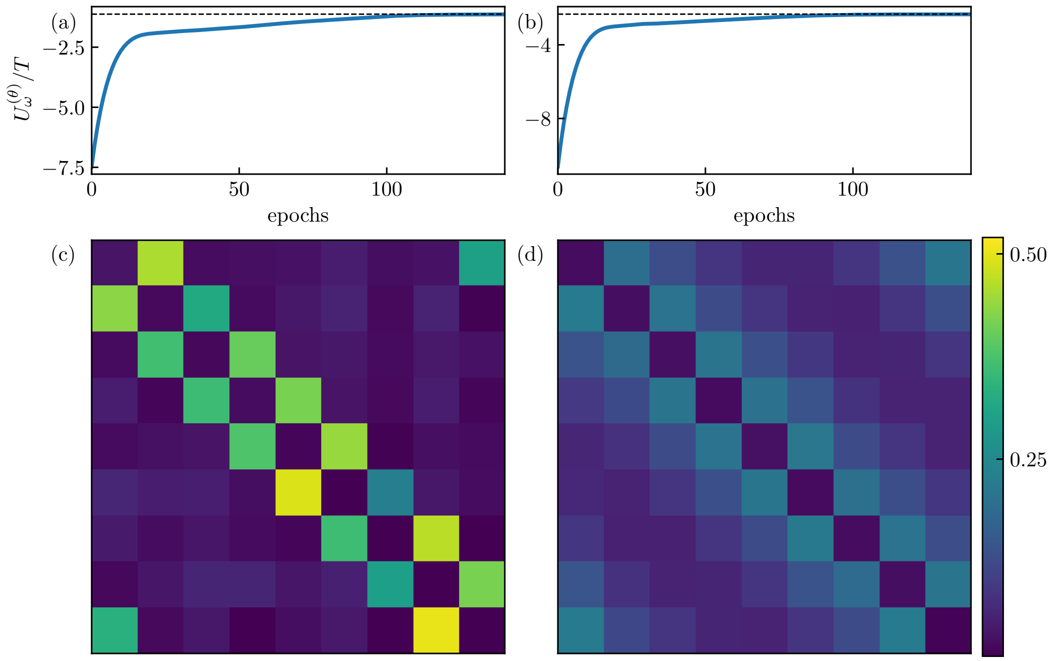

FIG. S6. (a) Training a transformer to maximize the log-likelihood with which it would generate a trajectory of a 9-site FA model. The horizontal black line denotes the value of the path weight associated with the original model. (c) Attention map averaged over all configurations in the trajectory for the original FA model. (b,d) Same as (a,c), but the model now includes long-range interactions per Eq. S4. Panels (a) and (b) show that the transformer has learned the original dynamics precisely; comparison of (c) and (d) shows that the transformer has learned that the two models possess local and nonlocal dynamical rules, respectively.

S3. LEARNING A NONLOCAL DYNAMICS

The dynamical rules in the lattice active matter model described in the main text and the FA model described in the previous section are local, depending only on nearest-neighbor particles. In this section we demonstrate that the transformer can likewise learn nonlocal dynamical rules, where rates depend on the states of distant particles. We first consider a variant of the FA model by adding long-range interactions to the rules shown in Table S1. The rate for flipping spin i in this model is given by

$$W_i = \sum_{j=0}^{L/2} \frac{(n_{i+j+1} + n_{i-j-1})}{2^j} \left[c(n_i - 1) + (1-c)n_i\right], \tag{S4}$$

where n_i is equal to one if the spin at site i is up and zero otherwise. We set $c = 0.1$ and $L = 9$, and generate trajectories of length $T = 10^6$. To show the transformer's ability to capture long-range interactions, we have used a minimal neural network consisting of just a single transformer layer with a single self-attention head. Consequently, all particle interactions must be captured in the single attention operation used in this network, because all other operations within the neural network work on the level of single-particle features (see Methods).

In Fig. S6(a) and (b) we show the result of training this minimal transformer on a trajectory of the original FA model and the nonlocal variant (S4) respectively. The learned dynamics are accurate for both models, confirmed by the fact that trajectory weights converge to the exact values associated with the original dynamics. We show the attention map learned by the minimal neural networks averaged over all particle configurations observed in the trajectory, for both the short-range and long-range models, in Fig. S6(c) and (d). These attention maps show that the transformer trained on a trajectory of the model with short-range interactions primarily considers nearest-neighbor interactions to determine the rates of spin flips, while the transformer trained on a model with long-range interactions assigns a larger relative importance to all particle interactions.

Finally, we consider an original dynamics containing a large number of distinct rates on account of a global conditioning applied to the trajectory. We consider the conditioned dynamics of the (short-ranged) FA model, whose trajectories are biased towards having atypical values of the activity. The generator of this model is given by

$$W_s^{\text{doob}} = \mathcal{L}[W_s - \theta(s)I]\mathcal{L}^{-1}, \tag{S5}$$

where W_s is obtained by multiplying the off-diagonal elements of the FA-model generator W by e^{-s}, $\theta(s)$ is the largest eigenvalue of W_s, and $\mathcal{L}$ is the corresponding left eigenvector of W_s as a diagonal matrix [52, 53]. Although the sets of forbidden and allowed transitions are the same

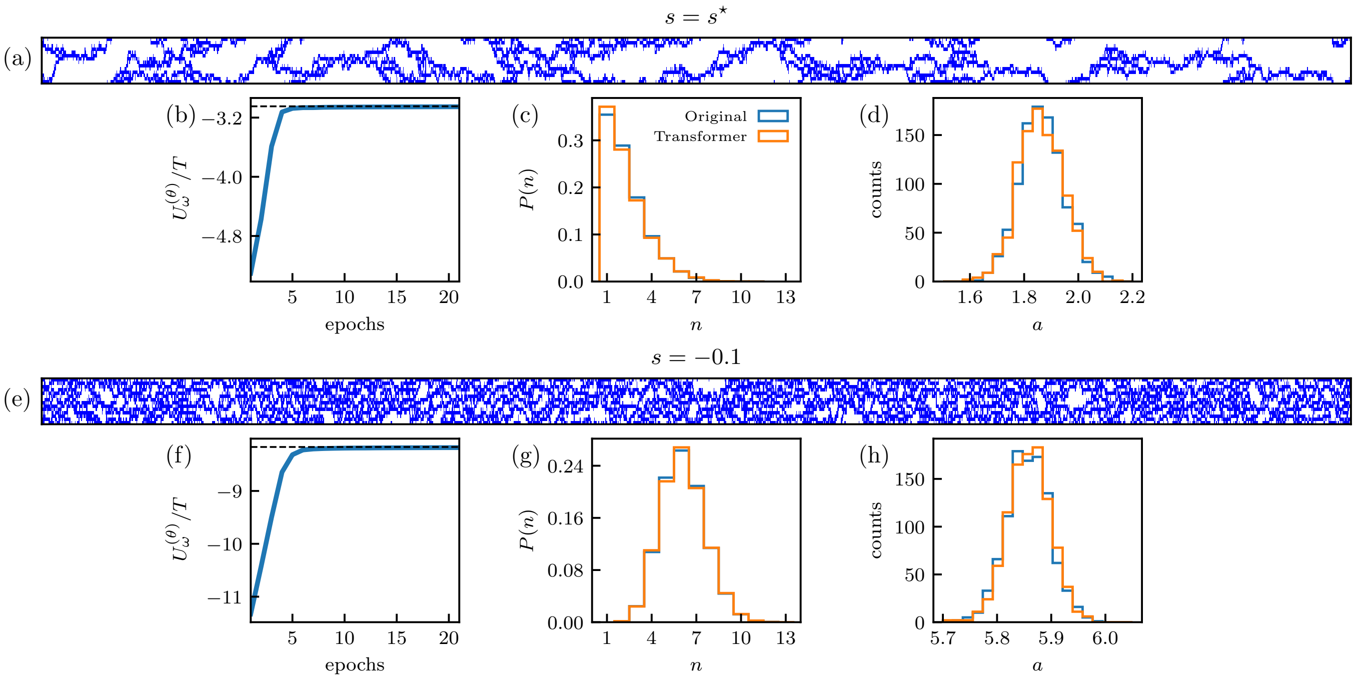

FIG. S7. Learning the long-ranged dynamics of the conditioned FA model. The dynamical rules of the conditioned generator are nonlocal and consist of a large number of distinct rates. (a) Trajectory of length $T = 2000$ generated by a transformer trained on a trajectory of the conditioned FA model with $N = 14$ lattice sites, $c = 0.3$, and $s = s^\star \approx 0.017$. Space is vertical, time is horizontal, and `1`s and `0`s are blue and white, respectively. (b) Optimization of a transformer to maximize the log-likelihood of the training trajectory. The horizontal black line denotes the value of the path weight associated with the original model. (c) Probability of the lattice containing n spins in state `1` during a trajectory of length $T = 10^6$, both for the original (exact conditioned) dynamics and the transformer dynamics. (d) Histogram of the activity k of 1000 trajectories of length $T = 10^4$. (e–h) As (a–d), but now for $s = -0.1$.

for both W and $W_s^{\rm doob}$, the transition rates of $W_s^{\rm doob}$ depend on the entire lattice configuration.

In Fig. S7, we show the result of training a transformer in Mode 1 on a trajectories of conditioned FA models of length $T = 10^6$ with $N = 14$ lattice sites. As values for the conditioning field, we use $s = s^\star \approx 0.017$ for which the susceptibility $\chi(s) = \theta''(s)$ is maximal for this lattice size, and $s = -0.1$. Typical trajectories of the conditioned dynamics for $s = -0.1$ have larger activity than typical trajectories of the unconditioned FA model. Trajectories generated by the trained transformers are shown in Figs. S7(a) and (e), and are markedly different compared to the unconditioned dynamics shown in Fig. S1(a). The rapid convergence of $U_\omega^{(\boldsymbol{\theta})}$ to its exact value for both values of s is shown in Figs. S7(b) and (f), and demonstrates the transformer's ability to correctly represent the long-range interactions of the conditioned FA model. In Figs. S7(c) and (g), we further validate this statement by measuring the probability of the lattice containing n up-spins over trajectories created with the original dynamics and by forward-propagating the transformer dynamics. In Figs. S7(d) and (h), we study the distribution of the activity for conditioned trajectories with both dynamics. The agreement with the exact conditioned dynamics is good.

SUPPLEMENTARY REFERENCES

[39] Fredrickson, G. & Andersen, H. C. Kinetic ising model of the glass transition. *Physical Review Letters* **53**, 1244–1247 (1984).

[40] Butler, S. & Harrowell, P. The origin of glassy dynamics in the 2d facilitated kinetic ising model. *The Journal of chemical physics* **95**, 4454–4465 (1991).

[41] Garrahan, J. P. & Chandler, D. Geometrical explanation and scaling of dynamical heterogeneities in glass forming systems. *Physical Review Letters* **89**, 035704 (2002).

[42] Whitelam, S., Selin, V., Park, S.-W. & Tamblyn, I. Correspondence between neuroevolution and gradient descent. *Nature communications* **12**, 6317 (2021).

[43] Bodineau, T., Lecomte, V. & Toninelli, C. Finite size scaling of the dynamical free-energy in a kinetically constrained model. *Journal of Statistical Physics* **147**, 1–17 (2012).

[44] Garrahan, J. P. Classical stochastic dynamics and continuous matrix product states: gauge transformations, conditioned and driven processes, and equivalence of trajectory ensembles. *Journal of Statistical Mechanics: Theory and Experiment* **2016**, 073208 (2016).

[45] Den Hollander, F. *Large Deviations*, vol. 14 (American Mathematical Soc., 2008).

[46] Touchette, H. The large deviation approach to statistical mechanics. *Physics Reports* **478**, 1–69 (2009).

[47] Whitelam, S., Jacobson, D. & Tamblyn, I. Evolutionary

reinforcement learning of dynamical large deviations. *The Journal of chemical physics* **153** (2020).

[48] Jacobson, D. & Whitelam, S. Direct evaluation of dynamical large-deviation rate functions using a variational ansatz. *Phys. Rev. E* **100**, 052139 (2019).

[49] Garrahan, J. P. Simple bounds on fluctuations and uncertainty relations for first-passage times of counting observables. *Physical Review E* **95**, 032134 (2017).

[50] Casert, C., Vieijra, T., Whitelam, S. & Tamblyn, I. Dynamical large deviations of two-dimensional kinetically constrained models using a neural-network state ansatz. *Physical Review Letters* **127**, 120602 (2021).

[51] Beltagy, I., Peters, M. E. & Cohan, A. Longformer: The long-document transformer. *arXiv preprint arXiv:2004.05150* (2020).

[52] Chetrite, R. & Touchette, H. Nonequilibrium Markov processes conditioned on large deviations. *Ann. Henri Poincaré* **16**, 2005–2057 (2015).

[53] Causer, L., Banuls, M. C. & Garrahan, J. P. Optimal sampling of dynamical large deviations via matrix product states. *Physical Review E* **103**, 062144 (2021).
